# Unexpected zinc dependency of ferroptosis: what is in a name?

**DOI:** 10.18632/oncotarget.27951

**Published:** 2021-06-08

**Authors:** Po-Han Chen, Jen-Tsan Chi

**Keywords:** ferroptosis, iron, zinc, ZIP7, HERPUD1

## Ferroptosis – an iron-dependent cell death

The importance of metal homeostasis for our health is illustrated by extensive disease phenotypes associated with their abnormal accumulation or deficiency. While the conventional role of bioactive metals is to serve as structural or catalytic cofactors, accumulating evidence has suggested additional signaling and regulatory roles. Among metals, zinc is an abundant trace element vital for many functions. For example, zinc is an essential component of more than 3000 zinc-finger transcription factors. Zinc is also a critical cofactor for over 300 diverse enzymes, including copper/zinc superoxide dismutase (CuZnSOD) and DNA repair proteins. In addition, zinc is appreciated to have a dynamic role as a “second messenger” in the glucose homeostasis, insulin signaling and diabetes [1]. Therefore, the levels of zinc may impact the function of a wide variety of proteins and biological processes, however, much remains unknown about other biological processes which are regulated by zinc.

Extreme oxidative stresses have been recognized to trigger cell death for decades, but the underlying mechanisms were poorly understood. Various inhibitors of cystine importer xCT, such as erastin, can block cystine import and trigger oxidative stress-induced death in certain cancer cells. An important insight is gleaned when the erastin-induced death can be protected by iron chelators and the protection effects can be reversed only by Fe^2+^, but not Cu^2+^, Mn^2+^, Ni^2+^, or Co^2+^ [2]. Therefore, the term “ferroptosis” has been coined to highlight iron-dependency as the cardinal feature of this form of regulated death [2]. Consistently, elevated cellular iron sensitized cells to ferroptosis. Furthermore, many ferroptosis determinants are found to regulate ferroptosis via affecting the cellular iron levels [3]. Even iron level is established as a critical trigger for ferroptosis, the mechanisms remain incompletely clear. One popular hypothesis is that iron drives the non-enzymatic Fenton reaction and amplifies the generation of reactive oxygen species (ROS) [4]. However, experimental proof for such hypothesis is still limited.

## Unexpected role of zinc and ZIP7 in ferroptosis

Recently, we found that zinc, similar to iron, was also essential for ferroptosis [5]. Zinc chelator (TPEN) potently antagonize ferroptosis, and zinc supplementation can trigger ferroptosis protected by iron chelators [5]. Interestingly, zinc was not included in the panel of metals tested in the original study [2]. To further investigate the role of zinc in ferroptosis, we identified ZIP7, a member of Zrt- and Irt-like protein family that regulates the cytosolic zinc transport from endoplasmic reticulum (ER), was essential for ferroptosis. Chemical or genetic inhibition of ZIP7 robustly protected cells from ferroptosis. Since ZIP7 mediates the translocation of zinc from ER to cytosol, we speculate ZIP7 inhibition may inhibit ferroptosis by blocking the zinc translocation. Furthermore, ZIP7 depletion strongly induced the expression of several genes known in ferroptosis (xCT, ATF3, HSP5, and DDIT3). Among these induced genes, the induced HERPUD1 was responsible for the ferroptosis protection mediated by ZIP7 depletion [5]. Together, these data strongly indicate the unexpected essential role of zinc and ZIP7 in regulating ferroptosis through organellar communication.

Previous studies proposed low level of zinc acts as an antioxidant via regulating glutathione, enzymes (i.e., CuZnSOD), iron-mediated lipid peroxidation and iron-binding to phospholipids [6]. However, other experimental data suggested that zinc and zinc transporters promote ferroptosis. For example, zinc can enhance mitochondrial ROS generation [7], induce lipid peroxidation and trigger ferroptosis via the phosphorylation of xCT [8]. Consistent with our results, “iron-free” zinc oxide nanoparticles also trigger ferroptosis via increasing ROS, lipid oxidation, and depleting glutathione [9]. While it is still possible that zinc affect ferroptosis through regulating iron levels, multiple independent studies on the essential role of zinc in ferroptosis may prompt re-examinations of the central role of iron in ferroptosis as implied in the name of this form of regulated cell death.

Other than ZIP7, another member of zinc transporter, ZIP14, was also reported to be essential for ferroptosis as its deletion in liver can rescue ferroptosis-induced liver fibrosis [10]. In addition to ZIP family of transporters, some iron transporters, such as ferroportin-1, also exports zinc [11]. Therefore, whether the zinc transport function of ferroportin-1 contributes to its regulation of ferroptosis need to be carefully considered. Furthermore, given zinc cannot trigger Fenton reaction to amplify ROS, it is possible that zinc, or even iron, may promote ferroptosis via mechanisms unrelated to Fenton reaction.

## Implication of zinc for the ferroptosis biology

To further elucidate the role of zinc vs. iron in ferroptosis, it is obvious much more works remain to be accomplished. First, it will be critical to determine the dynamics and movement of intracellular zinc during ferroptosis. As both ZIP7 [5] and ZIP14 [10] are essential for ferroptosis, the transportation of zinc between different cellular compartments during ferroptosis may provide important insights. Second, it is critical to identify the zinc-regulated proteins and biological processes that affect ferroptosis. For example, metallothioneins (MTs) protein family is known to chelate zinc and protect cells from oxidative stress. MT-1G has been shown to protect cells from ferroptosis [12], possibly through regulating zinc level. The zinc finger E-box-binding homeobox 1 (ZEB1), a EMT regulator, is recognized to promote ferroptosis [13]. In addition, zinc finger protein 367 (ZNF367) promotes metastasis by activating YAP/TAZ [14]. As metastatic cancer cells are particularly sensitive to ferroptosis [15] and YAP/TAZ promoted ferroptosis [16, 17], it is possible that zinc may regulate ZNF367 to promote metastasis and ferroptosis through YAP/TAZ. Third, it will be important to define the roles of zinc during physiological and pathological conditions that trigger ferroptosis. For example, zinc is elevated during the brain and heart ischemia [18] in which ferroptosis may contribute to the neuronal death. Therefore, zinc elevation may contribute to the neuronal death of ischemia injury via ferroptosis related mechanism.

The importance and therapeutic potential of ferroptosis has gained much recognition in the past decade. The discovery of unexpected role of zinc in ferroptosis will give us the chance to re-examine previous experimental data to dissect the role of zinc vs. iron during ferroptosis. In addition, the ferroptosis promotion by zinc may help to explain the occurrence of ferroptosis without iron accumulation. Furthermore, various compounds that interfere with zinc function or transport may block ferroptosis and have therapeutic potential for many human diseases involving ferroptosis. Together, these additional insights will further understandings and therapeutic deployment of ferroptosis to improve human diseases.
